# Competing risk of death and end-stage renal disease in incident chronic kidney disease (stages 3 to 5): the EPIRAN community-based study

**DOI:** 10.1186/s12882-016-0379-3

**Published:** 2016-11-15

**Authors:** Carole Ayav, Jean-Baptiste Beuscart, Serge Briançon, Alain Duhamel, Luc Frimat, Michèle Kessler

**Affiliations:** 1 INSERM, CIC-EC 1433, Nancy, France; 2 Pôle S2R, Epidemiology and clinical evaluation, University Hospital, Vandoeuvre-les-Nancy, France; 3 Geriatric Department, University Hospital, Lille, France; 4 Department of Biostatistics, UDSL, Lille, EA2694 France; 5 Lorraine University, Paris Descartes University, Apemac, Nancy, EA4360 France; 6 Department of Nephrology, University Hospital, Vandœuvre-les-Nancy, France

**Keywords:** Chronic kidney disease, Competing-risk analysis, Epidemiological study, Incidence, Outcomes, Risk factors

## Abstract

**Background:**

Although chronic kidney disease (CKD) affects a growing number of people, epidemiologic data on incident CKD in the general population are scarce. Screening strategies to increase early CKD detection have been developed.

**Methods:**

From a community-based sample of 4,409 individuals residing in a well-defined geographical area, we determined the number of patients having a first serum creatinine value ≥1.7 mg/dL and present for at least 3 months that allowed us to calculate an annual incidence rate of CKD (stages 3 to 5). CKD (stages 3 to 5) was defined by estimated glomerular filtration rate (eGFR) <60 mL/min/1.73 m^2^. We also described the primary care, outcomes and risk factors associated with outcomes using competing risks analyses for these CKD patients.

**Results:**

A total of 631 incident CKD patients (stages 3 to 5) were followed-up until the occurrence of death and dialysis initiation for more than 3 years. The annual incidence rate of CKD (stages 3 to 5) was estimated at 977.7 per million inhabitants. Analyses were performed on 514 patients with available medical data. During the study, 155 patients (30.2 %) were referred to a nephrologist, 193 (37.5 %) died and 58 (11.3 %) reached end-stage renal disease and initiated dialysis. A total of 139 patients (27.6 %) had a fast decline of their renal function, 92 (18.3 %) a moderate decline and the 272 remaining patients had a physiological decline (21.1 %) or a small improvement of their renal function (33.0 %). Predictors of death found in both Cox and Fine-Gray multivariable regression models included age at diagnosis, anemia, active neoplasia and chronic heart failure, but not a low glomerular filtration rate (GFR). Age at diagnosis, anemia and a low GFR were independently associated with dialysis initiation in Cox model, but anemia was not found to be a risk factor for dialysis initiation in Fine-Gray model.

**Conclusions:**

This large cohort study provided useful epidemiological data on incident CKD (stages 3 to 5) and stressed the need to improve the hands-on implementation of clinical practice guidelines for the evaluation and the management of CKD in primary care.

## Background

Chronic kidney disease (CKD) is a common condition and the increased incidence of end-stage renal disease (ESRD) is a growing public health challenge worldwide [1–6]. Less than 2 % of CKD patients in all stages except stage 4 progress to ESRD and undergo renal replacement therapy (RRT) [7]. CKD is associated with a markedly increased risk of cardiovascular morbidity and mortality and it is clear that the majority of CKD patients, especially those aged 75 and over, dies before reaching ESRD largely due to worsening heart disease [7–12].

The early identification of patients at increased risk for CKD or in the early stages is therefore critical and could facilitate timely therapeutic interventions to slow both progression of CKD to ESRD and cardiovascular diseases with benefits on both kidney and patient survival [13, 14]. Thus screening strategies to increase early recognition of individuals at risk were developed implementing surveillance of elevated plasma creatinine, microalbuminuria, and more recently routine reporting of estimated glomerular filtration rate (eGFR) in a high-risk target population [3, 15–22]. Family general practitioners (GPs) are responsible for CKD screening and timely referral to nephrologist. This creates opportunity for coordinated and optimal management of CKD to reduce morbidity and mortality [23].

Epidemiological data on incident CKD in the general population are the only ones permitting non-biased prognostic studies to be carried out, but they are scarce [5]. A recent literature review highlighted substantial heterogeneity between prevalence studies related to a wide range of study designs, criteria used for the selection of patients, and methods for measuring renal function [2]. Among the 43 selected studies, 6 high-quality studies allowed an estimate of the prevalence in a range of 1.7 to 8.1 %.

The objectives of the present community-based study was to determine the number of patients residing in a well-defined geographical area having a first serum creatinine value ≥1.7 mg/dL after January 1^st^, 2004 and still present for at least 3 months, and to describe their primary care, outcomes and risk factors associated with outcomes using competing risks analyses.

## Methods

### Study design

EPIRAN (EPidémiologie de l’Insuffisance Rénale chronique dans l’Agglomération Nancéienne) was a community-based epidemiological study based on active and passive data collection. The EPIRAN study was a prospective observational cohort study. As a result, the assignment of a human participant to a particular health-related intervention was not designed in advance by a trial protocol but fell within current clinical practice.

The study was conducted in the general population from a well-defined geographical area, the Urban Community of Greater Nancy (UCGN). This area is located in Eastern France and comprises a population of 258,000 inhabitants according to the 1999 census; it has a University Hospital and 2 private clinics.

### Study population

Included patients, called incident CKD patients (stages 3 to 5), were all adult patients (≥18 years) residing in the given geographical area and having a first serum creatinine value ≥1.7 mg/dL reached after January 1^st^, 2004 and present for at least 3 months.

### Data collection

Our study used laboratory data from routine tests. All 16 clinical laboratories gave their agreement to participate and were asked to report electronic data on patients fulfilling the inclusion criteria. As eGFR was not automatically generated by laboratories in France in 2004, its assessment could not be used as a diagnostic criterion.

In the absence of patient’s objection, validation of data was conducted on 50 000 dosages that had been declared in two and a half years. To avoid duplication between laboratories, double-entries were systematically checked first. Then previous creatinine levels were screened for each patient in order to identify prevalent CKD patients. When the first high creatinine measurement was not confirmed after a 3-month period we systematically searched for a creatinine level <1.7 mg/dL. At the end of this process, all patients with a creatinine level ≥ 1.7 mg/dL prior to January 1^st^, 2004, or a level lowered back to below 1.7 mg/dL were excluded. Only creatinine values ≥1.7 mg/dL were systematically reported by laboratories in the UCGN from January 1^st^, 2004 to June 30^th^, 2006.

An expert committee composed of nephrologists and epidemiologists critically reviewed and validated all exclusions and inclusions, and confirmed the persistence of kidney injury for all included patients, using a standardized process.

Each prescribing physician was contacted by a study nurse to give additional information on registration status. Each GP was asked about history of kidney disease and nephrology care. However, some GPs refused to provide any data about their patients. Consequently, 117 patients had missing data for all covariates except age, sex, and creatinine level, and were excluded in statistical analyses of secondary objectives.

Clinical and biochemical characteristics were collected. eGFR was calculated using the simplified Modification of the Diet in Renal Disease (MDRD) formula for patients with creatinine level ≥1.7 mg/dL [24]. CKD (stages 3 to 5) was defined solely by eGFR <60 mL/minute/1.73 m^2^. The number of included patients allowed us to calculate an annual incidence rate of CKD (stages 3 to 5).

As the study was aimed to observe the management of patients in real life, and therefore based on the strict respect of usual GP’s medical practice, the only visits and assessments collected by the study nurses were those carried out and requested by GPs. GPs were therefore free to schedule visits, order additional laboratory testing, and refer their patients to nephrologists.

Patients were followed-up until the occurrence of death, dialysis initiation, move outside the study area, or the end of the study (date of point on December 31^st^, 2009).

Progression of CKD during the study period was classified as following: improvement of eGFR if the difference between the final and initial values of serum creatinine (fSC-iSC) was ≤0, physiological decline if 0 < fSC-iSC ≤ 2, moderate decline if 2 < fSC-iSC < 5 and fast decline if fSC-iSC ≥ 5 [25].

Data about nephrology care, dialysis initiation and kidney transplant were collected for all included patients from all three nephrology departments located in the UCGN and from the French Renal Epidemiology and Information Network (REIN) registry [26]. To identify deaths among all included patients, the national register of deaths was interrogated at the end of the follow-up period [27].

### Statistical analyses

The annual incidence rate of CKD (stages 3 to 5) in UCGN was estimated from the whole study cohort (*N =* 631). It was calculated using the general population estimates for a health service catchment area as the denominator, based on population estimates from the 1999 Census [28]. Over the first decade of 2000, this population was unchanged [28]. Incidence rates according to gender and age were also calculated. Standardized incidence rate was also calculated according to direct standardization method and using WHO European standard population as reference [29].

Statistical analyses were performed on the 514 patients who had available characteristics*.* Baseline characteristics were presented in terms of mean and standard deviation (STD) for continuous variables, and expressed as frequency and percentage for categorical variables.

#### Univariable and multivariable analyses

Death and dialysis initiation were considered as competing risks, whereas other events were censored. The crude cumulative incidence functions were estimated for death and dialysis initiation using the method of Kalbfleisch and Prentice [30, 31]. We used two different models for uni- and multivariable analyses for the survival regression models on the whole cohort: i) a Cox proportional hazard regression model on the cause-specific hazards of death and of dialysis initiation, ii) a Fine-Gray regression model on the sub-distribution hazards of death and of dialysis initiation [32, 33].

#### Management of missing data

Given a large number of missing biological data, Hb level was the only variable taken into account. There was 11 % of missing Hb values. Values for covariates with missing values were obtained by multiple imputations using the MICE package as recommended for a Cox proportional hazard model analysis [34]. This was achieved with regression switching imputation using linear or logistic regression models dependingon the nature of the incomplete covariate fitted [34, 35]. This procedure was repeated five times to obtain five draws for each missing value in five distinct datasets.

Covariates were selected in the multivariable analysis using a stepwise procedure adapted to multiple imputation methodology [36]. According to Rubin’s approach, the coefficients and variances obtained with the final model on each imputed dataset were averaged by taking into account the intra-variance of the model and inter-variance between the imputed datasets [37].

#### Log-linearity assumption

The log-linear assumption of the Cox proportional hazard model was assessed using the Martingale residuals [38]. Since the log-linearity assumption was violated for age, hemoglobin (Hb) level, and eGFR, they were transformed into categorical variables. The cut-off values were identified with graphic investigations using Martingale residuals plots, maximization of the Gray test, and on the basis of medical expertise and consensus.

#### Sensitivity analysis

The cut-off value for eGFR was mainly determined by its association with the risk of hemodialysis which could lead to an underestimation of the relationship between eGFR and the risk of death. A sensitivity analysis was conducted to explore the relationship between the risk of death and various transformations of eGFR: continuous, logarithmic, or every possible cut-off of the variable.

#### Softwares

All statistical calculations were performed on SAS© v 9.2 (SAS Institute Inc., Cary, NC, USA) using the R statistical programming language and computing environment with survival, cmprsk, and Mice packages [39].

## Results

**Fig. 1:**
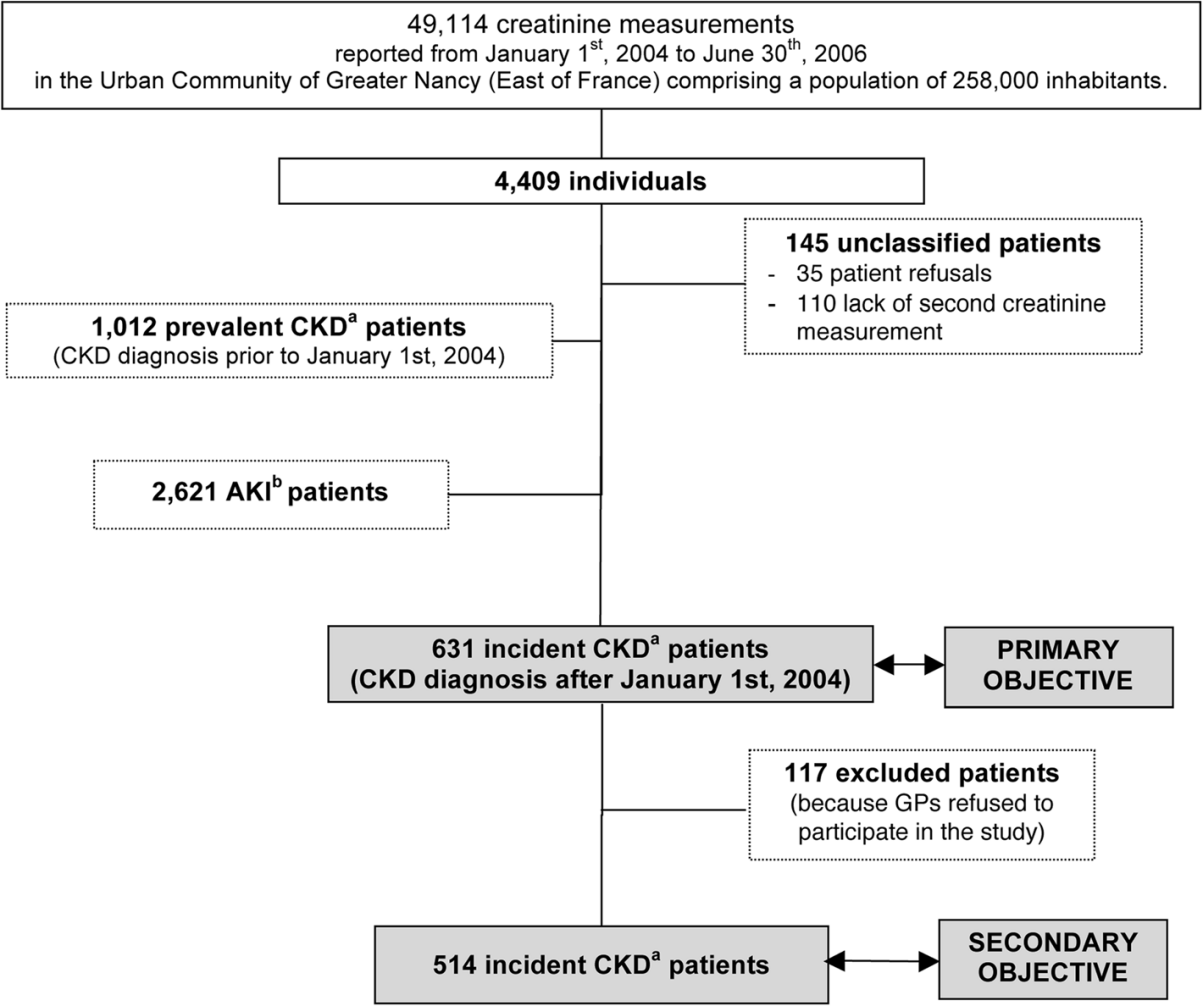
Flow diagram of patient distribution according to their status. ^a^ Chronic kidney disease; ^b^ Acute kidney injury

A total of 49,114 creatinine values ≥1.7 mg/dL were reported in the UCGN from January 1^st^, 2004 to June 30^th^, 2006. Among the 4,409 individuals, 35 patients refused to be enrolled and 110 were excluded because of lack of a second creatinine measurement; 1,012 patients with a serum creatinine ≥1.7 mg/dL reported prior to January 1^st^ 2004 were considered as prevalent CKD patients; 2,621 patients had an acute deterioration of renal function, and creatinine level lowered back to below 1.7 mg/dL during the follow-up; finally, 631 were diagnosed incident CKD patients after validation by the expert committee (Fig. 1).

The first serum creatinine measurement with a value ≥1.7 mg/dL was initiated by a GP for 45.0 % of the 631 cases, a non nephrologist specialist for 49.5 %, and a nephrologist for only 5.5 %. Reasons why a specialist ordered a creatinine blood test were mostly a standard medical checkup on planned admission to hospital (57.8 %), on emergency visit (15.7 %), on pre-anesthesia assessment (12.8 %) and a routine follow-up for a chronic condition (13.7 %). A period of time was needed before confirmation of diagnosis by a second creatinine determination, and was similar for GPs and non nephrologist specialists (7 ± 4.2 and 6.4 ± 4.3 months, respectively).

### Incidence rate of CKD (stages 3 to 5)

The annual incidence rate of CKD was estimated from the whole cohort (*N =* 631) at 977.7 per million population (pmp) [902.9–1,057.0], approximately two-fold higher in men than in women (1,338.2 [1,211.5–1,474.6] and 654.9 pmp [571.7–746.7], respectively). Standardized incidence according to the WHO population-type was 767.1 pmp [764.0–770.1].

**Fig. 2:**
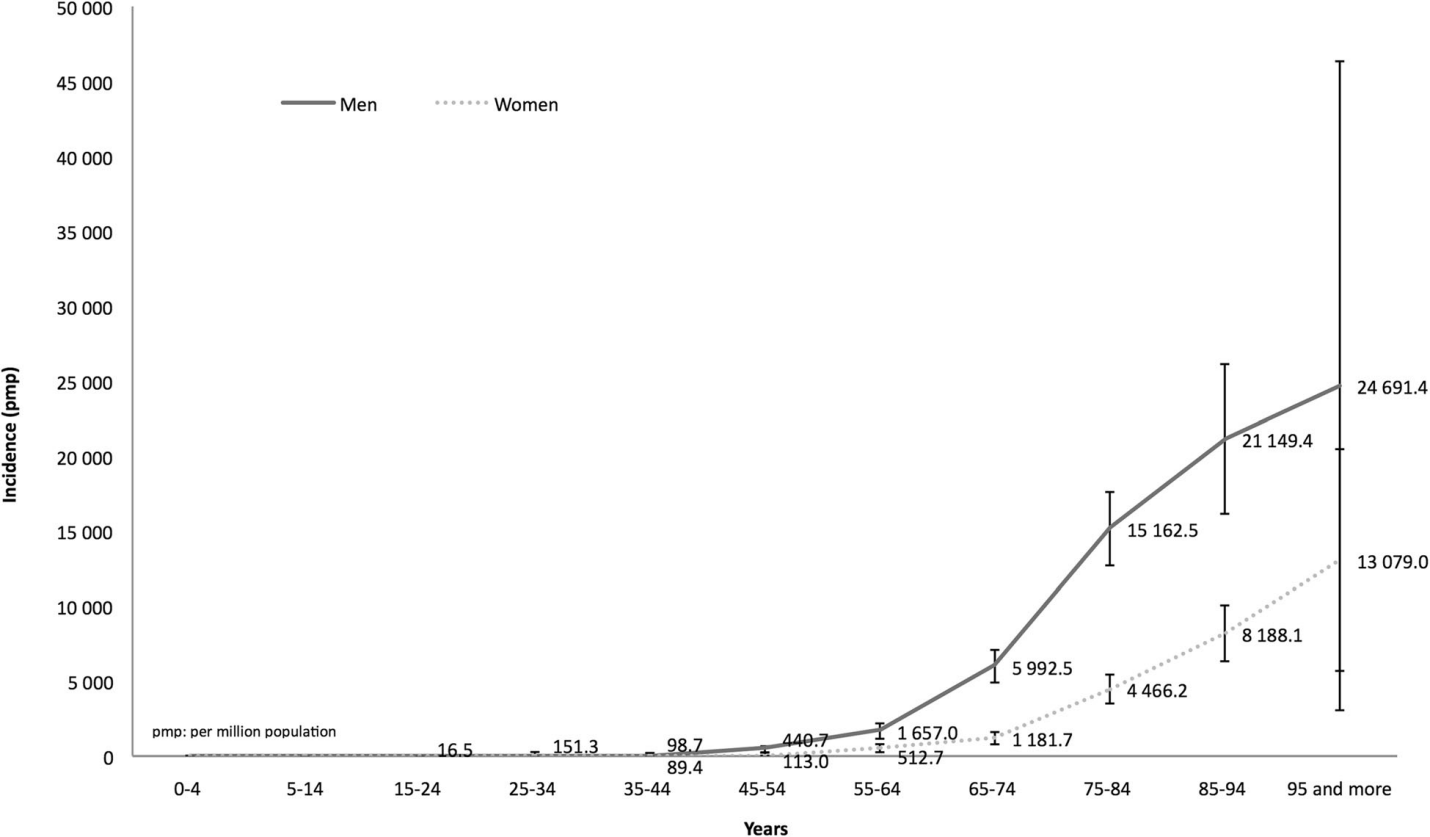
Cumulative incidence of chronic kidney disease according to age at diagnosis and gender. pmp: per million population

Incidence rate of CKD was very low under age 45 and increased exponentially after age 55 (mean age: 76.4 ± 12.8 years), with a male preponderance regardless of age (Fig. 2). The sex ratio male/female (1.83) decreased progressively with age from 3.89 for patients aged 55–65 to 1.89 for patients aged >95.

### Baseline characteristics

As some GPs refused to participate in the study, medical data were available for only 514 patients and used for the secondary objective (Fig. 1). We compared demographic characteristics of CKD patients with and without additional medical data and the only difference concerned age: 75.95 ± 13.05 *vs.* 78.52 ± 11.56 years, respectively *p =* 0.0467. Baseline characteristics of the 514 patients are displayed in Table 1. The majority of patients (73.9 %) were aged ≥70 years at the time of diagnosis and 65.4 % were men.

**Table 1:** Baseline characteristics of incident CKD patients with available data (*N =* 514)

	Missing data (%)	N	%	Mean	STD	Median	Q1	Q3	Min	Max
Age (year)	0	514		76.0	13.0	77.7	69.6	84.9	17.5	99.9
< 70		134	26.1							
70–80		156	30.4							
80–85		96	18.7							
≥ 85		128	24.9							
Gender	0									
Men		336	65.4							
Women		178	34.6							
BMI (kg/m^2^)	36.9	324		27.1	5.4	26.1	23.7	30.1	14.7	47.5
≤ 20		21	6.5							
]20–25]		97	29.9							
]25–30]		122	37.7							
> 30		84	25.9							
SBP (mm Hg)	36.4	327		136.8	22.8	135	120	150	78	240
DBP (mm Hg)	36.4	327		73.3	12.6	70	66	80	38	120
Number of comorbidities	1.3	507		2.9	1.3	3	2	4	0	7
0		2	0.4							
1		54	10.7							
2		144	28.4							
3		155	30.6							
4		152	30.0							
Cardiovascular diseases		436	86.0							
Diabetes mellitus		173	34.1							
Ischemic cardiomyopathy		159	31.4							
Heart failure (NYHA III or IV)		117	23.1							
Hypertension		368	72.6							
Stroke		45	8.9							
Lower limb peripheral arterial disease		101	19.9							
Neoplasia		87	17.2							
Hemoglobin (g/dL)	11.3	454		12.1	2.1	12.1	10.6	13.5	5.4	18.4
Serum creatinine (mg/dL)	0	514		2.2	1.3	1.9	1.8	2.1	1.7	23.7
eGFR (mL/min/1.73 m^2^)	0	514		32.0	8.6	32.4	27.3	38.9	1.7	52.8
< 15		25	4.9							
15–29		168	32.7							
30–44		314	61.1							
45–59		7	1.4							

*BMI* body mass index, *DBP* diastolic blood pressure, *eGFR* estimated glomerular filtration rate using MDRD formula (Modification of the Diet in Renal Disease), *Min* minimum, *Max* maximum, *Q1* quartile 1, *Q2* quartile 2, *STD* standard deviation, *SDB* systolic blood pressure

About one-third of patients were diabetic and one-quarter had congestive heart failure (CHF). Estimated GFR was 32 ± 8.6 mL/min/1.73 m^2^. All patients were CKD diagnosed at stage 3 (61.1 %), stage 4 (32.7 %) and stage 5 (6.2 %). At inclusion, Hb level was 12.1 ± 2.1 g/dL; more than half of the patients were anemic (Hb level <12 g/dL for women, <13 g/dL for men). Proteinuria was measured in only 9.7 % of patients (*n =* 50). The mean value for proteinuria was 2.1 ± 2.8 g/24 h (median value: 1.2 g/24 h). At the time of the first serum creatinine value > 1.7 mg/dL, 15 % of patients had already attended a nephrologist consultation, and 19.6 % of patients had had a serum creatinine measurement as part of their follow-up care for a known nephropathy.

### Outcomes

Patients were followed-up for more than three years (37.9 ± 24.0 months). During the course of the study, a total of 155 patients (30.2 %) were referred to a nephrologist within 16.0 ± 16.2 months after being included, 193 (37.5 %) died and 58 (11.3 %) reached ESRD and initiated dialysis (no preemptive kidney transplantation).

**Fig. 3:**
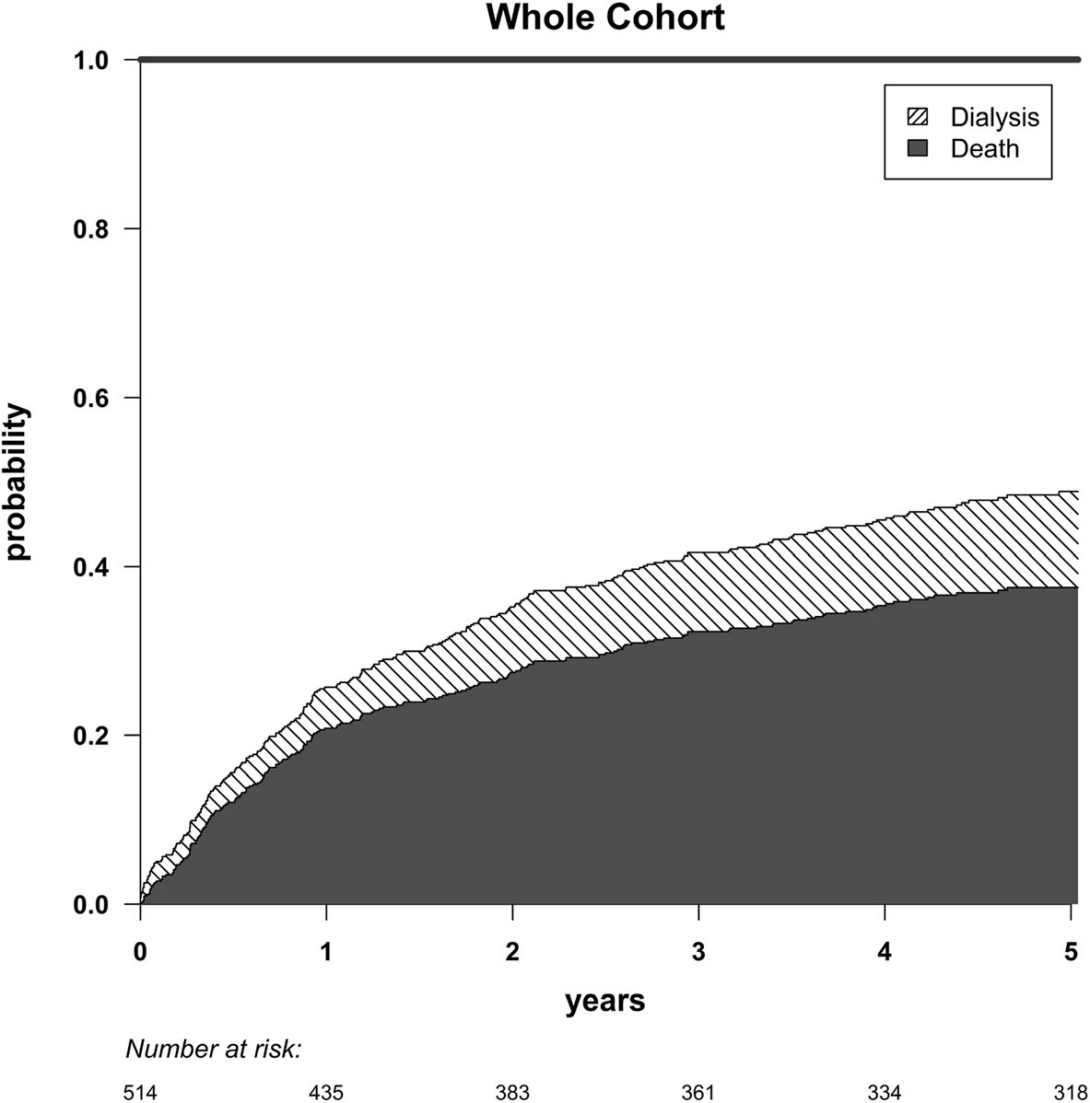
Cumulative incidence of death and dialysis initiation during the follow-up. The cumulative incidence curves are stacked

Among those who died, only 17.1 % were referred to a nephrologist. The leading causes of death were: cardiovascular diseases (32.3 %), cancer (28.6 %), other or unknown (15.3 %) and infectious diseases (8.5 %). The five-year probabilities of death and dialysis initiation reached 33 % and 12 %, respectively (Fig. 3).

A total of 139 patients (27.6 %) had a fast decline of their renal function, 92 (18.3 %) a moderate decline and the 272 remaining patients had a physiological decline (21.1 %) or a small improvement of their renal function (33 %) (Table 2). Fast progressors were more likely to reach ESRD or death (*p <* 0.0001).

**Table 2:** Progression of CKD according to the renal function during the study period (*N =* 503)

Annual progression of CKD	Patient’s condition at the end of the study			
N (%)	Without ESRD	ESRD	Death	Total
Improvement of renal function^a^	96 (57.8 %)	12 (7.2 %)	58 (35 %)	166
Physiological decline^b^	74 (69.8 %)	5 (4.7 %)	27 (25.5 %)	106
Moderate decline^c^	48 (52.2 %)	12 (13 %)	32 (34.8 %)	92
Rapid decline^d^	39 (28.1 %)	29 (20.9 %)	71 (51.0 %)	139
Total	257	58	188	503

*CKD* chronic kidney disease, *ESRD* end-stage renal disease

^a^if the difference between the final and initial values of serum creatinine (fSC-iSC) was ≤0

^b^if 0 < fSC-iSC ≤ 2

^c^if 2 < fSC-iSC < 5

^d^if fSC-iSC ≥ 5

### Competing risk analyses of death and dialysis initiation

Variables associated with death using Cox univariable regression analysis were age at diagnosis, Hb level <11 g/dL, active neoplasia and CHF (Table 3).

**Table 3:** Risk factors associated with death and dialysis initiation (univariable analyses)

	Cox model				Fine-Gray model			
Variables	Death		Dialysis initiation		Death		Dialysis initiation	
	HR	95 % CI	HR	95 % CI	HR	95 % CI	HR	95 % CI
Age (years)								
< 70	1.00		1.00		1.00		1.00	
70–80	1.91	[1.23 –2.97]	0.44	[0.23 –0.84]	2.07	[1.34 –3.22]	0 .39	[0.21 –0.75]
80–85	1.87	[1.15 –3.03]	0.30	[0.13 –0.74]	2.06	[1.28 –3.33]	0 .27	[0.11 –0.66]
> 85	2.78	[1.8 –4.32]	0.45	[0.22 –0.92]	2.98	[1.92 –4.62]	0.36	[0.17 –0.73]
Gender								
Male	1.00		1.00		1.00		1.00	
Female	1.13	[0.84 –1.52]	1.37	[0.81 –2.32]	1.10	[0.82 –1.49]	1.27	[0.75 –2.15]
Hemoglobin								
≥ 11 g/dL	1.00		1.00		1.00		1.00	
< 11 g/dL	2.37	[1.76 –3.17]	2.52	[1.44 –4.41]	2.13	[1.59 –2.86]	1.89	[1.09 –3.29]
Cancer < 5 years								
No	1.00		1.00		1.00		1.00	
Yes	1.67	[1.18 –2.35]	0.95	[0.45 –2.01]	1.62	[1.14 –2.32]	0.78	[0.37 –1.67]
Chronic heart failure								
No	1.00		1.00		1.00		1.00	
Yes	1.74	[1.29 –2.36]	0.68	[0.33 –1.38]	1.78	[1.32 –2.4]	0.59	[0.29 –1.19]
Ischemic cardiopathy								
No	1.00		1.00		1.00		1.00	
Yes	1.28	[0.95 –1.72]	0.80	[0.44 –1.43]	1.30	[0.97 –1.74]	0.75	[0.42 –1.34]
Stroke								
No	1.00		1.00		1.00		1.00	
Yes	1.45	[0.94 –2.25]	0.57	[0.18 –1.84]	1.48	[0.96 –2.3]	0.52	[0.16 –1.68]
Peripheral arterial disease								
No	1.00		1.00		1.00		1.00	
Yes	1.07	[0.76 –1.52]	1.55	[0.87 –2.75]	1.03	[0.73 –1.44]	1.59	[0.9 –2.81]
Diabetes								
No	1.00		1.00		1.00		1.00	
Yes	0.92	[0.68 –1.25]	2.54	[1.51 –4.28]	0.88	[0.65 –1.18]	2.62	[1.56 –4.41]
High blood pressure								
No	1.00		1.00		1.00		1.00	
Yes	0.82	[0.6 –1.11]	0.85	[0.48 –1.5]	0.85	[0.62 –1.15]	0.90	[0.51 –1.58]
Others								
No	1.00		1.00		1.00		1.00	
Yes	1.56	[0.96 –2.53]	1.18	[0.54 –2.61]	1.54	[0.94 –2.52]	1.08	[0.49 –2.39]
eGFR								
> 20 mL/min	1.00		1.00		1.00		1.00	
< 20 mL/min	1.30	[0.77 –2.2]	6.93	[3.96 –12.13]	0.85	[0.49 –1.48]	6.04	[3.39 –10.74]

X variables entered into the multivariable analyses

*CI* confidence interval, *eGFR* estimated glomerular filtration rate using MDRD formula (Modification of the Diet in Renal Disease), *HR* hazard ratio

**Fig. 4:**
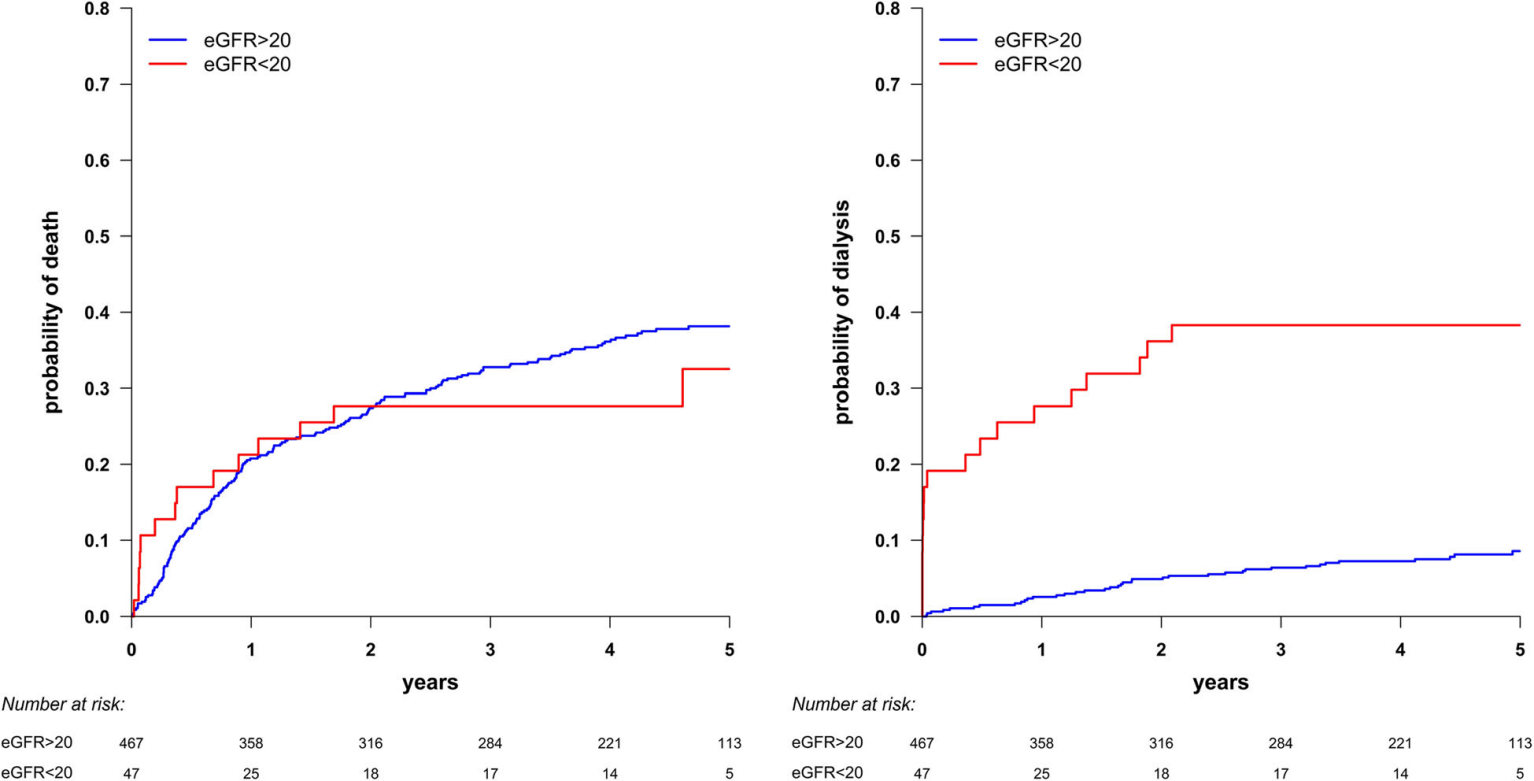
Cumulative incidence of death (Panel **a**: on the left side) and end-stage renal disease (ESRD) (Panel **b**: on the right side) in patients with eGFR <20 mL/min/1.73 m^2^ (solid line) and other patients (dotted line). HD: hemodialysis; eGFR: estimated glomerular filtration rate (using MDRD*- modification of diet in renal disease*-formula)

They remained independently associated with death in the Cox multivariable regression analysis (Table 4). No other variables associated with death as those identified in the Cox model were significant in the Fine-Gray model (Table 4). eGFR was not found to be a risk factor for death in the main analysis (Table 4, Fig. 4-Panel a) and in the sensitivity analysis.

**Table 4:** Risk factors associated with death and dialysis initiation (multivariable analyses). Covariates were selected in the multivariable analysis using a stepwise procedure adapted to multiple imputation methodology

	Death					Dialysis			
	Cox model		Fine-Gray model			Cox model		Fine-Gray model	
	HR	95 % CI	HR	95 % CI		HR	95 % CI	HR	95 % CI
Age (years)					Age (yrs)				
< 70	1.00		1.00		< 70	1.00		1.00	
70 –80	1.85	[1.19 –2.89]	2.07	[1.32 –3.23]	70 –80	0.34	[0.17 –0.65]	0.30	[0.16 –0.58]
80 –85	1.78	[1.09 –2.91]	2.03	[1.24 –3.31]	80 –85	0.33	[0.14 –0.81]	0.33	[0.14 –0.78]
> 85	2.56	[1.64 –4.00]	2.84	[1.82 –4.43]	> 85	0.53	[0.25 –1.10]	0.40	[0.18 –0.88]
Hemoglobin					Hemoglobin				
≥ 11 g/dL	1.00		1.00		≥ 11 g/dL	1.00		1.00	
< 11 g/dL	2.18	[1.61 –2.93]	2.01	[1.49 –2.73]	< 11 g/dL	2.05	[1.16 –3.60]	1.62	[0.92 –2.84]
Cancer < 5 years					eGFR				
No	1.00		1.00		≥ 20 mL/min	1.00		1.00	
Yes	1.61	[1.14 –2.28]	1.62	[1.1 –2.37]	< 20 mL/min	8.13	[4.37 –15.13]	8.32	[4.4 –15.71]
Chronic Heart Failure					Diabetes				
No	1.00		1.00		No	1.00		1.00	
Yes	1.72	[1.26 –2.34]	1.81	[1.31 –2.49]	Yes	3.41	[1.92 –6.07]	3.43	[1.93 –6.09]
Stroke									
No	1.00		1.00						
Yes	1.46	[0.93 –2.28]	1.44	[0.89 –2.32]					
High Blood Pressure									
No	1.00		1.00						
Yes	0.76	[0.55 –1.04]	0.80	[0.58 –1.11]					

*CI* confidence interval, *HR* hazard ratio

Variables associated with dialysis initiation using Cox univariable regression analysis were: age at diagnosis, Hb level <11 g/dL, diabetes, and eGFR <20 mL/min (Table 3). In the Cox multivariable regression analysis, age at diagnosis, Hb level <11 g/dL, and eGFR <20 mL/min remained independently associated with dialysis initiation (Table 3, Fig. 4-Panel b). No other variables associated with dialysis initiation as those identified in the Cox model were significant in the Fine-Gray univariable analysis. In the Fine-Gray multivariable model, Hb level <11 g/dL was not found to be a risk factor for dialysis initiation (Table 4).

## Discussion

In the population-based EPIRAN study, we detected patients having two consecutive serum creatinine values ≥1.7 mg/dL over a three-month period. Each case was critically reviewed by an expert committee. The annual incidence rate of CKD (stage 3 to 5 CKD defined as eGFR < 60 ml/min/1.73 m^2^/year) was estimated to 977.7 pmp using routine blood testing systematically reported by clinical laboratories of an urban territory with a population of 250,000. The incidence was two-fold higher in men than in women. This result is not surprising given the fact that the same cut-off was used in both genders. The identified patients were elderly with multiple comorbidities. Our results were close to that previously observed [40]. Studies conducted to define incidence for CKD are scarce, most of studies evaluate its prevalence [3–5, 40]. Prevalence studies indicate CKD burden whereas incidence studies contribute to the implementation of longitudinal follow-up and the monitoring of CKD progression, and may therefore suggest prevention strategies.

One of the major strengths of our study is the innovative nature of the methodology based on repeated measurement of serum creatinine to confirm the persistence of renal injury. Firstly, it is worth noting that the systematic and automated self-reporting of abnormal creatinine levels above the cut-off value by laboratories may help primary care physicians detect more accurately patients at increased risk for CKD or in early stages, particularly among elderly [16, 22, 41, 42]. Secondly, asserting incidence and chronicity of the condition is of major interest to differentiate CKD from acute kidney injury (AKI) and not misclassify patients with an acute deterioration as chronic. Nearly 60 % of patients with at least one increased value in creatinine level above 1.7 mg/dL had creatinine level under this cut-off value during the course of our study. All medical reports were appraised by an expert committee including nephrologists and epidemiologists. Study nurses systematically interviewed all clinical laboratories in order to track all measures under the cut-off value that would not have been reported. This work allowed us to assert the chronicity of renal disease. Thirdly, the large sample size and the prospective non interventional design with a longitudinal follow-up add to the robustness of our findings reflecting real life. Finally, competing risks analyses showed that most patients died prior to the stage of ESRD and allowed us to identify predictive factors associated to death and dialysis initiation. Age at diagnosis, presence of anemia, CHF and active neoplasia were independent risk factors for all-cause mortality. Our results are consistent with those previously reported [7]. Anemia develops as a frequent complication of CKD, and can have a deleterious effect not only on progression of CKD but also on progression of cardiovascular diseases and events [1]. In our study, 45.9 % of incident CKD patients (stages 3 to 5) experienced decline of their renal function during the course of the study, and those with a fast decline were more likely to reach ESRD or death, as previously shown among prevalent CKD patients [10, 43]. In addition, eGFR was not associated with risk of death that was inconsistent with previous findings [10, 44]. Go et al found non linear relations between the GFR and the risk of death [10]. In 38,520 non-dialysis-dependent CKD patients, each 5 mL/min/1.73 m^2^ decline in eGFR was associated with higher risk of death due to cardiovascular disease (hazard ratio: 1.10; 95 % confidence interval: 1.08 to 1.12) [44]. The first possible explanation for our contradictory results is the selection of incident patients whereas those previous studies included prevalent patients. All the published data that correlated eGFR with death were observed in studies performed in prevalent CKD patients. Prevalent CKD patients are different from incident CKD patients because of a survival bias due to the non-inclusion of patients who have died before the study starts and to the fact that development of CKD increases the risk of death. A second explanation is supported by clinical characteristics of patients included in the EPIRAN study; they were mostly stage 3 CKD patients at the time of diagnosis and non progressors (54.1 %). From our point of view, progression of CKD played a major role when it was found to be associated with risk of death in prevalent patients. Finally, a completely different explanation could be supported by recent findings. Mandelli et al. assessed mortality prediction in the oldest old (aged 85 years and older) with five different equations to estimate GFR They showed that risk of mortality was significantly higher for reduced GFR estimated with the Berlin Initiative Study-1 and Cockroft-Gault equations over the short time, whereas the MDRD formula appeared a more consistent predictor after five years [45]. However, a significantly higher risk of death after 5 years was found in the oldest old with a very low eGFR (<30 mL/min/1.73 m^2^), irrespective of the equation. In addition, we found that independent risk factors significantly associated with dialysis initiation were age at diagnosis, presence of diabetes and GFR below 20 mL/min/1.73 m^2^. Interestingly, elderly (>70 years) were less likely to undergo dialysis. This may be due to a bias towards nephrology referral and indication for dialysis, a low likelihood of experiencing progression of CKD to ESRD in elderly or a patient choice [46].

CKD patients identified in our cohort were mainly stages 3-4 primary care patients, but the first abnormal creatinine level (above the cut-off value) was measured in a public or private hospital for 42.8 % of patients. For serum creatinine measurements ordered by a non nephrologist specialist, they were done mostly within a standard medical checkup that was not systematically transmitted to the family GP, and as a result, delayed CKD diagnosis. Clinical inertia in response to CKD diagnosis was seen in primary care but also with specialist care, whether they worked in healthcare institutions or not. The same phenomenon was observed for the check up to diagnose CKD. Proteinuria was sought only in 7.9 % of patients despite French recommendations for diagnosis and management of CKD and international guidelines advocating systematic screening for markers of kidney damage such as proteinuria [47–50]. Proteinuria is predictive for CKD progression but also for progression to ESRD [5, 48, 51–55]. Proteinuria was also shown to impact on the occurrence of cardiovascular events [56]. Reduction of proteinuria is therefore a major determinant for reducing the risk of CKD progression to ESRD and cardiovascular events [49, 57–61]. Our study confirm that early identification of CKD patients on the basis of presence of reduced eGFR or proteinuria is desirable to permit early intervention for reducing risk of cardiovascular events, progression to kidney failure or death [62]. Over the past decade, the therapeutic arsenal to reduce CKD burden, such as prevention of complications, slowing progression, and prevention of cardiovascular risk, has markedly expanded [48, 50, 63]. However, practice patterns suggest that physicians remain unfamiliar with recommendations for detection and management of CKD as highlighted in several studies [64–67]. The lack of acute awareness of clinical practice guidelines for CKD varies according to the type of registration status (nephrologist > specialist > GP) [65, 66]. As early detection is within the realm of the family GPs, they should feel more comfortable with making the initial diagnosis of CKD and providing appropriate primary and ongoing care to patients.

Regarding medical monitoring of patients, our findings demonstrated that only 28.3 % of patients were referred to a nephrologist after CKD diagnosis. There is probably a bias in nephrologist referral related to the age of patients. It is indeed possible that GPs chose not to refer elderly to nephrologists because 1) they felt that elderly could not be candidates for RRT, and 2) reduced GFR remained relatively stable in most of them. Several recent studies showed the benefit of conservative management in elderly as long as they remained asymptomatic [68]. A prospective, community-based study provided a compelling evidence for a causal relationship between pre-ESRD medical care and outcomes [69].

A recent literature review focusing on studies regarding changes in kidney function over time highlighted the importance of incorporating the rate of kidney decline in the staging of CKD for better risk stratification on mortality and cardiovascular outcomes for individual patients as CKD is a dynamic [70]. Although most CKD patients will die from cardiovascular disease rather than progress to kidney failure, it is of interest to develop prevention programs for patients who will progress to ESRD, such as tertiary preventive care and regular monitoring to detect early patients most likely to progress. This will let appropriate referral of patients who might benefit from a nephrology service while allowing the majority to remain in primary care, and prevent that local nephrology services become overwhelmed [71]. Nevertheless, it is mandatory to conduct medical surveillance in those remaining in primary care, and avoid all situations (iatrogenic and not) which may lead to worsening of renal function or decompensate.

The following limitations should be considered. First, we used a single creatinine cut-off value as inclusion criterion to define impaired renal function because French clinical laboratories did not calculate eGFR when we designed the study. Although French clinical laboratories were required to report eGFR for each creatinine value in 2005, we could not change this main inclusion criterion during the study. The creatinine cut-off value of 1.7 mg/dL was selected on the basis of the only published incidence study when the EPIRAN study was designed [40]. Drey et al. used this value as recommended by the Renal Association [72]. CKD diagnosis could only be assigned to patients who had had serum creatinine measurement, and more specifically those who had repeated measurement. As a result, incidence of CKD may be underestimated, because only patients with serum creatinine repeated measurement were included in our study. However, this cut-off value allowed to detect a majority of new patients having stage 3 CKD and avoid misclassification of healthy elderly people with eGFR <60 mL/min. Second, because of the non interventional design of our study, only patients who were prescribed creatinine measurement could be identified. Another limitation was the scarcity of proteinuria determination, although presence of proteinuria was recognized to predispose to ESRD, even with a normal renal function [3, 5, 13, 55, 57, 59, 60]. Our results were consistent with previous findings highlighting that special attention must be paid to improve albuminuria screening [3, 5]. Despite these limitations, a non interventional approach is the only possible way of studying management of patients in real life.

## Conclusion

In conclusion, our study provided annual incidence rate of CKD stage 3 to 5 in a community-based population, confirmed that death was far more likely than advancement to RRT in CKD patients, identified predictors of death and dialysis initiation, and highlighted the need to improve the hands-on implementation of guidelines for the evaluation and management of CKD in primary care.

## Abbreviations

AKI: Acute kidney injury
BMI: Body mass index
CHF: Congestive heart failure
CI: Confidence interval
CKD: Chronic kidney disease
DBP: Diastolic blood pressure
eGFR: Estimated glomerular filtration rate
ESRD: End-stage renal disease
GP: General physician
Hb: Hemoglobin
HR: Hazard ratio
Max: Maximum
MDRD formula: Simplified modification of the diet in renal disease
Min: Minimum
pmp: Per million population
Q1: Quartile 1
Q2: Quartile 2
REIN registry: French Renal Epidemiology and Information Network registry
RRT: Renal replacement therapy
SBP: Systolic blood pressure
SCf-SCi: Difference between the initial and final values of serum creatinine
STD: Standard deviation
UCGN: Urban Community of Greater Nancy
